# Two distinct populations of Bovine IL-17^+^ T-cells can be induced and WC1^+^IL-17^+^γδ T-cells are effective killers of protozoan parasites

**DOI:** 10.1038/srep05431

**Published:** 2014-06-25

**Authors:** R. K. Peckham, R. Brill, D. S. Foster, A. L. Bowen, J. A. Leigh, T. J. Coffey, R. J. Flynn

**Affiliations:** 1 School of Veterinary Medicine and Science, Sutton Bonington Campus, University of Nottingham, LE12 5RD

## Abstract

IL-17 has emerged as a key player in the immune system, exhibiting roles in protection from infectious diseases and promoting inflammation in autoimmunity. Initially thought to be CD4 T-cell-derived, the sources of IL-17 are now known to be varied and belong to both the innate and adaptive arms of the immune system. Mechanisms for inducing IL-17 production in lymphoid cells are thought to rely on appropriate antigenic stimulation in the context of TGF-β1, IL-6 and/or IL-1β. Using culture protocols adapted from human studies, we have effectively induced both bovine CD4^+^ and WC1^+^ γδ T-cells to produce IL-17 termed Th17 and γδ17 cells, respectively. The negative regulatory effect of IFN-γ on mouse and human IL-17 production can be extended to the bovine model, as addition of IFN-γ decreases IL-17 production in both cell types. Furthermore we show that infection with the protozoan *Neospora caninum* will induce fibroblasts to secrete pro-IL-17 factors thereby inducing a γδ17 phenotype that preferentially kills infected target cells. Our study identifies two T-cell sources of IL-17, and is the first to demonstrate a protective effect of IL-17^+^ T-cells in ruminants. Our findings offer further opportunities for future adjuvants or vaccines which could benefit from inducing these responses.

IL-17, a major pro-inflammatory cytokine, has been shown to have several cellular sources indicating a multitude of roles with the immune system, including causing both pathology and providing protection^1^. Th17 cells, a key producer of IL-17, have been tightly linked to the outcome of multiple parasite infections, including the protozoan *Toxoplasma gondii* where IL-17 has been shown to dominate central nervous system (CNS) pathology during chronic infection^2^. Moreover Th17 cells are known to be negatively regulated by IFN-γ and not to produce IL-17 and IFN-γ simultaneously. Aside from CD4 T-cells, γδ T-cells have been described as a major source of IL-17 including during infection with *Plasmodium berghei*^3^ and *Leishmania major*^4^. *Neospora caninum*, a closely related intracellular protozoan, causes abortion in cattle worldwide and economic losses due to depressed production yields^5^. Timing of infection dictates the pathological outcome; *de novo* infection of pregnant animals in the 1^st^ trimester leads to reabsorbed foetuses, during the 2^nd^ trimester abortion can occur and in the 3^rd^ trimester unborn calves can be congenitally infected leading to vertical transmission of the disease^5^. Studies have implicated IFN-γ in pathology but others report conflicting results suggesting both a protective, preventing abortion^6^, and a pathological, causing abortion, role^7^,^8^. This leads us to hypothesize that a source(s) of IL-17 may have an important role in protection against foetal death and host tissue damage either in isolation or combination with other cytokines.

A recent study has implicated a “cytokine storm” effect within placental tissues around the time of abortion^9^. Furthermore, we have recently shown that parasite limiting macrophages provoke IL-17 producing CD4 T-cells, with a Th17 phenotype equivalent to that seen in murine and human studies^10^. This is especially pertinent given the opposing effects that IL-17 and IFN-γ can have on each other. Given this, our hypothesis of IL-17 in protection against *N. caninum* and the abundance of γδ T-cells in young cattle in comparison to other mammals^11^, it was timely to investigate the ability of specific T-cell subsets to produce IL-17 and their functional relevance to protect against *N. caninum* infection.

## Results

### CD4 T-cells produce IL-17 under TCR and cytokine stimulation

**Figure 1:**
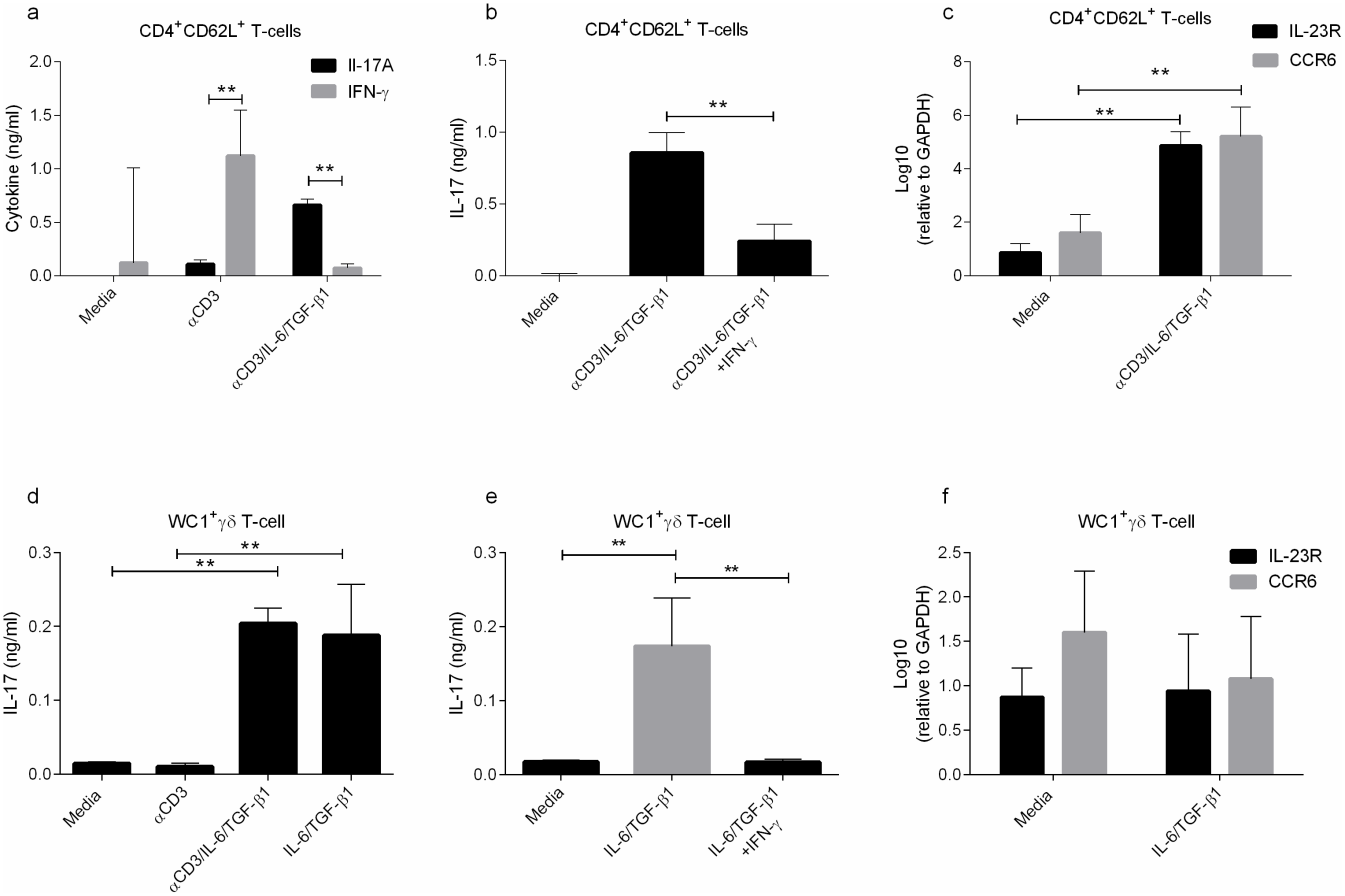
(a) 2.5 × 10^5^ Naïve CD4^+^CD62L^+^ T-cells were isolated and stimulated for 72 hrs with or without anti-CD3 (1 µg/ml) in the presence of IL-6 (40 ng/ml)/TGF-β1 (2 ng/ml) and tested for IL-17 and IFN-γ, (b) IL-17 was measured in cultures of T-cells stimulated as above that were grown in the presence of IFN-γ (20 ng/ml), (c) CCR6 and IL-23R RNA was measured in cells as cultured in (a). (d) 2.5 × 10^5^ WC1^+^ γδ T-cells were isolated and cultured with IL-6/TGF-β1, as above, with or without anti-CD3 and IL-17 was measured after 72 hrs. (e) γδ T-cells were cultured as in (d) with added IFN-γ (20 ng/ml) before measurement of IL-17. (f) CCR6 and IL-23R RNA was measured in γδ T-cells from (d) above. Data are means of triplicates ± SD and representative of one of four animals tested. Data was analysed using one way ANOVA (*P = <0.05, **P = <0.01). Data shown is representative of four independent experiments.

To test the concept that the cytokines, IL-6 and TGFβ1, can condition naïve bovine CD4^+^CD62L^+^ T-cells to differentiate into *de novo* IL-17 producing Th17 cells, naïve cells were stimulated in the presence of cytokines and TCR ligation by anti-CD3 for 72 hrs. The range of cytokine concentrations initially tested were IL-6 5 ng/ml–50 ng/ml and TGF-β1 2 ng/ml–16 ng/ml. Supernatants were tested for IL-17 production, which was found to correlate with increasing concentrations of IL-6 but not TGF-β1 (data not shown). The optimal concentration for maximal IL-17 production was 40 ng/ml of IL-6 and 2 ng/ml of TGF-β1 and, in line with previous findings, no IFN-γ could be detected in these cultures (Figure 1a). The addition of recombinant IFN-γ resulted in decreased IL-17 production (Figure 1b). Furthermore, these cells demonstrated elevated levels of *CCR6* and *IL-23R* transcripts, consistent with the Th17 phenotype (Figure 1c).

### γδ T-cells respond to cytokine stimuli but not TLR2 stimuli with IL-17 induction

γδ T-cells have been shown in both humans and mice to express IL-17 under various conditions. Using the above optimised IL-6/TGF-β1 concentrations above, cells were cultured both with and without anti-CD3 (Figure 1d). The results demonstrate that even in the absence of TCR ligation, cytokine stimulation is sufficient to induce IL-17^+^ WC1^+^γδ T-cells which we term γδ17 cells. Similar to our findings with CD4 T-cells, high levels of IL-17 production were consistent with little to no IFN-γ demonstrating cellular polarisation (data not shown). Likewise when IFN-γ was added to IL-6/TGF-β1 stimulated γδ T-cell cultures IL-17 production was found to be down-regulated (Figure 1e). Furthermore, γδ17 cells showed no increase in expression of *IL-23R* or *CCR6* transcripts when tested (Figure 1f).

Murine γδ T-cells have been known to induce IL-17 under TLR2 stimulation^12^. To test this in our system cells were cultured with Pam3CSK4, PGN or heat-killed *Staphylococcus aureus*; no IL-17 was induced under these conditions (data not shown).

### Fibroblasts infected with Neospora caninum are susceptible to killing by γδ17 T-cells

**Figure 2:**
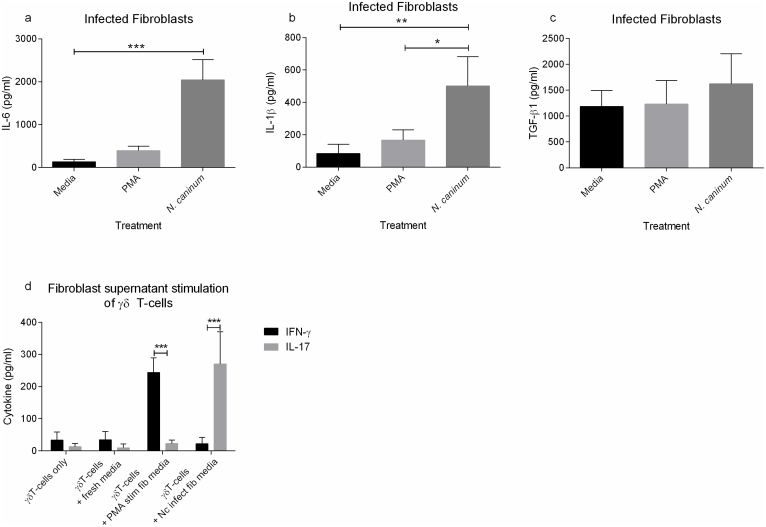
2 × 10^5^ Fibroblasts were infected with *N. caninum* MOI 1, PMA (25 ng/ml) or RPMI media and incubated for 72 hours. (a) IL-6 and (b) IL-1β and (c) TGF-β were measured using ELISA. (d) Supernatants were collected from fibroblasts as stimulated above and added to 2.5 × 10^5^ freshly isolated naïve γδ T-cells for 72 hrs thereafter IL-17 and IFN-γ was measured in supernatants. Treatments were fresh unconditioned media, supernatant from PMA-stimulated fibroblasts (PMA stim fib media), and media taken from *N. caninum* infected fibroblasts (Nc infect fib media). Data shown is mean of triplicates ± SD of a single representative animal from four tested. Data was analysed using one-way ANOVA (a–c) and 2-way ANOVA (d), *P = <0.05, **P = <0.01, ***P = <0.001. Data shown is representative of four independent experiments.

*N. caninum* transitions from rapidly dividing tachyzoites to slow growing bradyzoites, which preferentially form in muscle and CNS tissue, under pressure from host immunity. Should *N. caninum* recrudesce it is likely they will begin to multiply within tissue fibroblasts. Investigations into the cytokine response of naïve fibroblasts to infection with *N. caninum* found that, after 72 hrs, there was a striking and significant increase in production of IL-6, IL-1β but not TGF-β1 (Figure 2a–c). We found no evidence for secretion of IL-10, IL-4 or IFN-γ (data not shown). This demonstrates differential production of cytokines capable of driving IL-17 responses. To further test this, we added the supernatants derived from *N. caninum* infected fibroblasts to naïve γδ T-cells and tested the resulting supernatants for IL-17 and IFN-γ. Our results demonstrate that *N. caninum* conditioned fibroblast supernatants are capable of driving γδ17 T-cells with little to no IFN-γ production (Figure 2d).

**Figure 3:**
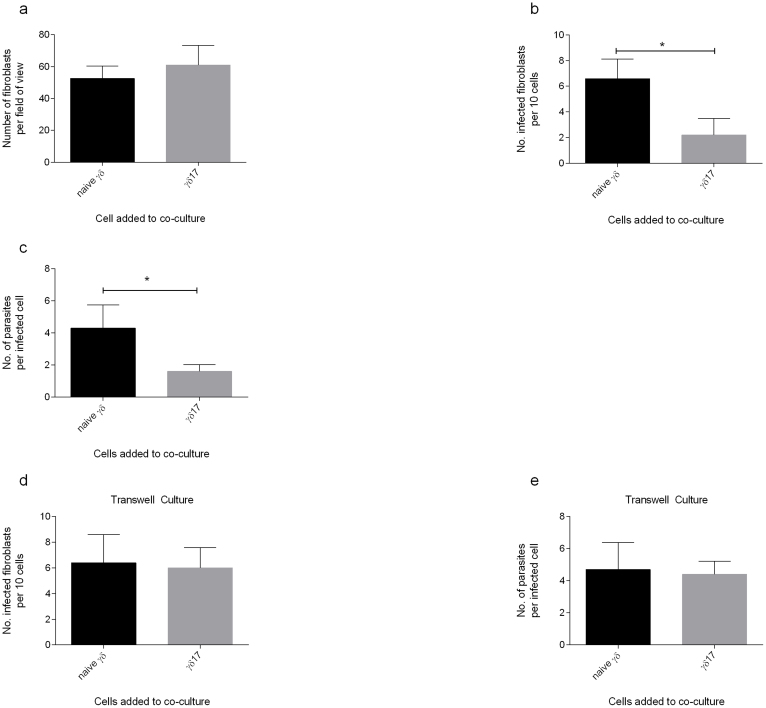
1 × 10^5^ Fibroblasts grown on cover slips were infected with *N. caninum* MOI 1, 4 hrs later 1 × 10^5^ autologous γδ T-cells conditioned to secrete IL-17 were added and cultures incubated for a further 24 hrs. (a) The number of fibroblasts per field of view was counted on a light microscope at 40×. (b) The average number of infected fibroblast was calculated from a 10 cell count and (c) the average number of parasites infecting each cell was recorded. For transwell experiments, transwell inserts were applied to infected or control fibroblast cultures before addition of γδ17 cells thereafter % infected cells (d) and number of parasites (e) were measured were measured. Data shown are means ± SD from 5 counts. Data shown is mean of triplicates ± SD of a single representative animal from four tested. Data was analysed using one-way ANOVA (*P = <0.05, **P = <0.01, ***P = <0.001, ns = not statistically significant). Data shown is representative of three independent experiments.

To determine if γδ17 T-cells are capable of killing infected host fibroblasts, fibroblasts were grown on coverslips, infected and incubated with naïve γδ cells or γδ17 cells for 24 hrs before microscopic examination (Figure 3a–d). The total number of fibroblasts was not significantly different between cultures incubated with unconditioned γδ and conditioned γδ17 cells (Figure 3a). When determining the percentage of infected cells per high powered field a significant decrease in infected fibroblasts cultured with γδ17 cells was observed (Figure 3b). Furthermore, examination of the number of parasites per cell revealed significant decreases between infected fibroblasts cultured with γδ and those cultured with γδ17 cells (Figure 3c). When co-cultures were conducted using transwell inserts, no significant differences in either percentage of infected cells (Figure 3d) or parasites/cell (Figure 3e) were found.

## Discussion

Herein we show that two separate populations of bovine T-cells are capable of inducing IL-17 expression under appropriate cytokine stimulation. The CD4 population retains features of human and mouse Th17 cells with high levels of *CCR6* and *IL-23R* expression. However, the bovine γδ17 cells produced are suggestive of a more transient, yet polarised, phenotype with no expression of *CCR6* or *IL-23R*. We have also shown that these cells can be stimulated by supernatants collected from *N. caninum*-infected fibroblasts. Furthermore, the resulting γδ17 cells show strong anti-parasite effects in a co-culture system that is dependent on cell-cell contact. One striking difference between our Th17 and γδ17 cells is in the expression of *CCR6* and *IL-23R.* The lack of IL-23R on γδ17 cells might indicate that these cells are not responsive to IL-23 and so do not undergo a stabilizing process similar to that reported in Th17 cells. The absence of CCR6 in our γδ17 may be explained by the differential use of homing chemokines dependent on the site of inflammation. Geherin *et al*^12^ demonstrated that skin homing ovine γδ T-cells expressing Il-17 use CCR6 but not their blood counterparts. This is relevant given that the γδ17 cells in our studies were generated from blood. Alternatively, this difference may be a reflection of the activation processes in both cell types. γδ17 cells were generated in the absence of TCR stimulation while Th17 cells were TCR cross-linked. This may also be reflective of their function *in vivo* during infection. As Th17 cells will most likely be activated within a lymph node they will need to migrate to the site of inflammation whereas γδ17 cells may be already present at the site of inflammation, thus can be activated solely by cytokines without requiring guidance to the site of inflammation. Indeed there is an abundance of γδ T-cells in bovine gut tissues during the early life period.

The role of Th17 cells during *N. caninum* infection could possibly be related to the generation of protective antibody or a higher level function such as orchestration of neutrophil/effector cell influx. During the course of infection with the closely related *T. gondii*, mice that display heightened Th17 responses show aggravated pathology following oral infection^13^ and during CNS infection/reactivation^14^. However, the impact of parasite strain and route of infection must be fully assessed as IL-17R^−/−^ mice show increased mortality rates due to reduced PMN recruitment^15^. Within *N. caninum* infection biology there already exists a contradictory role for IFN-γ where its function, or the outcome of its activity, would appear to be determined by timing of infection and thus timing of IFN-γ production. Our results suggest that IFN-γ can negatively regulate IL-17 production in two different T-cell types and thus may contribute to the suppression of protective or pathological responses. Certainly the γδ17 cells produced here would appear to be capable of mediating cell contact dependent immunity. Given the need for cell contact it would appear that γδ17 cells trigger cell autonomous killing of *N. caninum*, in an IFN-γ-independent mechanism. There is evidence to suggest that CD40-CD154 (CD40L) signaling can trigger autophagy in the absence of IFN-γ^16^. CD40 is expressed on fibroblasts^17^ and it is known that ConA stimulation causes the up-regulation of CD154 on bovine γδ T-cells^18^; however it remains to be determined if CD154 is expressed on bovine γδ17 cells.

Our findings raise the possibility that invoking IL-17 production either short or long term might provide protection against *N. caninum* infection, with bovine Th17 cells acting in the context of vaccination while γδ17 cells may act as a more short term or innate response to initial infection.

## Methods

### Parasite culture

*N. caninum* parasites (NcLiv), a gift from Professor Diana Williams University of Liverpool, were maintained in host VERO cells and harvested as previously described^10^.

### T-cell separation and culture

Healthy bovine donor tissues were used throughout and obtained in line with UK Home Office guidelines. CD4^+^CD62L^+^ cells were isolated in two steps as per Flynn & Marshall^10^. Briefly, anti-CD4 (AbD Serotec clone CC30, mouse IgG1) was used at a dilution of 1/50 to label 10^7^/ml PBMCs for 20 minutes. Cells were then labelled with secondary Miltenyi magnetic beads coasted with anti-mouse IgG1 as per manufacturer's instructions. The CD4^+^ fraction was then isolated using a positive selection protocol using an AutoMacs separator and collected into 1%BSA-PBS. Cells, 10^7^/ml, were subsequently labelled with anti-CD62L (AbD Serotec clone CC8 mouse IgG1) at 1/30 for 20 minutes and purified using the above secondary method. γδ T-cells were isolated in a single step using anti-WC1 (AbD Serotec Clone CC15), using the same indirect labelling protocol as above, anti-WC1 antibody was used at a concentration of 1/50 to label 10^7^ cells/ml. Cells were cultured in complete media [RPMI 1604 with 10% heat-inactivated foetal calf serum, 200 U/ml penicillin, 200 µg/ml streptomycin and 1% non-essential amino acids] with cytokines as described. Where α-CD3 stimulation is indicated plate bound stimulation was used, tissue culture plates were coated with antibody (VMRD – Clone MM1A) in sterile D-PBS (Sigma-Aldrich) overnight at 37°C at a concentration of 1 µg/ml. To induce IL-17 production, IL-6 and TGF-β1 were added to T-cell cultures at the indicated concentrations in the presence or absence of plate bound α-CD3. To polarize T-cells towards IL-17 secretion cells were cultured at a density of 2.5 × 10^5^/well in 24 well plates for 72 hrs, unless otherwise stated, with the indicated cytokines. All cultures were maintained at 37°C in 5% CO_2_ incubator.

### Fibroblast culture, infection and killing assays

Fibroblasts were isolated from livers of autologous blood donors according to Dobbs *et al*^19^, with minor modifications. To determine cytokine production following infection, fibroblasts [10^6^/ml] were grown in 48 well tissue culture plates and infection with freshly isolated parasites at a multiplicity of infection (MOI) of 1. Four hours after addition of parasites, the media was changed to remove extracellular parasites from the culture and cells were incubated for a further 72 hrs before collection of supernatant. To test the killing ability of γδ cells cultured to become IL-17 secreting (γδ17 cells), fibroblasts were grown on coverslips and infected as above. 4 hrs following infection γδ cells were added to fibroblasts at a ratio of 1:1. Cultures were incubated for 24 hrs before supernatants were removed, coverslips were rinsed in D-PBS, fixed in methanol and stained with Giemsa. Coverslips were examined under a Nikon YS2-H microscope to determine the number of fibroblasts per high powered field of view (HPV), number of infected fibroblasts per HPV and number of parasites per infected cell.

In some experiments transwell inserts, pore size 0.4 µm (Corning), were used to separate γδ cells from fibroblasts.

### ELISAs & Recombinant cytokines

Recombinant proteins used were as follows, bovine IL-6, bovine IFN-γ (Kingfisher Biotech) and human TGF-β1 (Peprotech). Recombinant proteins were re-suspended in complete media, see above, prior to use. ELISAs were used to quantify IL-17 (KingFisher Biotech), TGF-β1 (Promega), IL-6, IFN-γ, and IL-1β (Thermo-Scientific).

### Real-Time PCR

mRNA was isolated by Phenol-Chloroform extraction and reverse transcription performed with GoScript Reverse Transcription System (Promega). Samples were analysed, in quadruplicate, using an ABI 7900HT Real-time PCR system for levels of *IL-23R* (assay ID Bt03817892_m1) and *CCR6* (assay ID Bt04317064_s1) using Taqman assays (Applied Biosystems). Results are reported as expression levels, calculated using the Δct method, relative to *gapdh* (assay ID Bt03210913_g1).

### Statistical Analysis

Data was entered into GraphPad Prism software for statistical analysis using appropriate tests (see figure legends); *P* value of <0.05 was significant.

## Author Contributions

R.K.P., D.S.F., R.B., R.J.F. designed and conducted experiments; A.B. carried out real-time P.C.R. analysis; T.J.C., J.A.L., R.J.F. conceived the ideas for the study; R.J.F., T.J.C., J.A.L. drafted manuscript; all authors contributed to the final version of the manuscript.
